# Automated identification of dementia using medical imaging: a survey from a pattern classification perspective

**DOI:** 10.1007/s40708-015-0027-x

**Published:** 2015-12-21

**Authors:** Chuanchuan Zheng, Yong Xia, Yongsheng Pan, Jinhu Chen

**Affiliations:** 1 Shaanxi Key Lab of Speech & Image Information Processing (SAIIP), School of Computer Science and Engineering, Northwestern Polytechnical University, Xi’an, 710072 People’s Republic of China; 2 Centre for Multidisciplinary Convergence Computing (CMCC), School of Computer Science and Engineering, Northwestern Polytechnical University, Xi’an, 710072 People’s Republic of China; 3 Department of Radiation Oncology, Shandong Tumor Hospital, Jinan, 250117 People’s Republic of China

**Keywords:** Dementia, Computer-aided diagnosis, Medical imaging, Image processing, Feature extraction, Pattern classification

## Abstract

In this review paper, we summarized the automated dementia identification algorithms in the literature from a pattern classification perspective. Since most of those algorithms consist of both feature extraction and classification, we provide a survey on three categories of feature extraction methods, including the voxel-, vertex- and ROI-based ones, and four categories of classifiers, including the linear discriminant analysis, Bayes classifiers, support vector machines, and artificial neural networks. We also compare the reported performance of many recently published dementia identification algorithms. Our comparison shows that many algorithms can differentiate the Alzheimer’s disease (AD) from elderly normal with a largely satisfying accuracy, whereas distinguishing the mild cognitive impairment from AD or elderly normal still remains a major challenge.

## Introduction

Dementia is a chronic and progressive decline in cognitive function due to the damage or disease in the brain beyond what might be expected from normal aging [1]. There exist many varieties of dementia, among which the Alzheimer’s disease (AD) and frontotemporal dementia (FTD) are two of the most common types [2–4]. AD is the most prevalent dementia type, representing 60–80 % of the cases [5]. FTD was once considered rare, but it is now thought to account for up to 4 and 20 % of all dementia and memory disorders in clinics [6] and may be as common as AD among people younger than age 65 [7]. Other conditions that can also cause dementia include Creutzfeldt–Jakob disease (CJD), Huntington’s disease, Lewy body disease, Parkinson’s disease, vascular dementia, and Wernicke–Korsakoff syndrome.

Dementia is now a major global health and social threat. It was estimated that 35.6 million people worldwide were suffering from dementia in 2010 and the population was predicted to be doubled every 20 years as the world population ages [8, 9]. Due to the rapid increase of dementia cases, this disease has become an increasing death-factor around the whole world [10]. It is shown that, from 2000 to 2010, the deathrate of heart disease, breast cancer, prostate cancer, stroke, and HIV has dropped by 16, 2, 8, 23, and 42 %, respectively, whereas the deathrate of AD has increased by an astonishing 68 % [11]. Even worse, dementia generally presents a duration of more than 10 years after diagnosis [12], which may bring enormous impact and financial burden on individuals, families, health care systems, and societies as a whole [13–15].

The early symptoms of dementia include memory problems, difficulties in word finding and thinking processes, a lack of initiative, changes in personality or behavior, in day to day function at home, or at work, and in taking care of oneself. Some symptoms are reversible, whereas others are irreversible, depending upon the etiology of the disease. If the dementia can be diagnosed at its early stage, it is still possible to repair some reversible damages and thus slow down the process of irreversible damages, since evidences showed that the currently available medications for dementia, which can help people to maintain daily function and quality of life as well as stabilize cognitive decline, may be more beneficial if given early in the disease process. For instance, about 10–30 % of people with the mild cognitive impairment (MCI), which is usually thought to be the incubation of AD in clinical practice, develop to AD every year, whereas the conversion rate of normal aging group is just 1–3 % [16]. According to recent research, diagnosing MCI at its early stage and taking corresponding measures to protect certain neurological functions of patients will help to slow down the conversion from MCI to AD.

There exist some brief (5–15 min) tests that have reasonable reliability and can be used in the office or other settings to screen cognitive status for deficits which are considered pathological. Examples of such tests include the abbreviated mental test score (AMTS), mini-mental state examination (MMSE), modified mini-mental state examination (3MS) [17], cognitive abilities screening instrument (CASI) [18], and clock drawing test [19]. Although these tests can help diagnosing different types of dementia, they are generally recognized to be inadequate to classify the types of dementia at an early stage. Some people perform well on brief screening tests, but their memory and thinking impairments may be found with more comprehensive testing. Moreover, some tests have been shown to have educational, social, and cultural biases.

Medical imaging offers the ability to visualize degenerative histological changes, including the amyloid plaques, hypo-metabolism, and atrophy introduced by neurological disorders, which occur long before the neurodegenerative disorder is clinically detectable [20]. Hence, the widespread applications of medical imaging have led to a revolution in the early diagnosis of dementia [21–24]. The commonly used imaging modalities in dementia diagnosis include the magnetic resonance imaging (MRI), positron emission tomography (PET), and single-photon emission computed tomography (SPECT). Structural MRI uses a magnetic field and radio waves to create detailed images of the organs and tissues within human body and has been shown to be a surrogate for early diagnosis of AD, particularly in subjects clinically classified as amnestic MCI (aMCI) [25]. This technique offers several advantages, including greater availability, better soft tissue contrast, faster data acquisition, lower cost, and the possibility of automatically deriving quantitative indices of regional atrophy [26]. Accordingly, the validation of structure MRI as a marker of AD progression is the core project of the Alzheimer’s Disease Neuroimaging Initiative (ADNI). Functional PET with various radioactive tracers, e.g., 2-[^18^F]fluoro-2-deoxy-d-glucose (FDG) and ^18^C-Pittsburgh Compound (^11^C-PiB), can detect subtle changes in cerebral metabolism or amyloid deposition prior to anatomical changes are evident or a symptomatological diagnosis of probable dementia can be made with structure imaging [27–30]. Functional SPECT is similar to PET in its use of radioactive tracer material and detection of gamma rays. SPECT scans have low spatial resolution than PET scans, but are significantly less expensive. However, the interpretation of PET and SPECT images remains a challenge because the changes can be subtle in the early course of the disease and there can be some overlap with normal aging and other dementia types [31].

In medical imaging based dementia diagnosis, the acquired 3D images are still analyzed almost entirely through visual inspection on a slice-by-slice basis in search of familiar disease patterns. This requires a high degree of skill and concentration, and is time-consuming, expensive, and prone to operator bias. Thus, there is a strong demand for computer-aided automated dementia classification, which is expected to provide a useful “second opinion” and enable doctors to bypass the aforementioned issues. As a result, a great number of computer-aided automated dementia identification approaches have been proposed. The targets of those approaches are in threefold: (1) differentiating dementia cases from normal controls (NCs); (2) identifying different stages of dementia, such as separating MCI from AD cases; and (3) identifying different types of dementia, such as separating AD from FTD. There exist several publically available databases, including the Early Lung Cancer Action Program (ELCAP) [32], Open Access Series of Imaging Studies (OASIS) [33, 34], and Alzheimer’s disease Neuroimaging Initiative (ADNI) [35]. These databases have been broadly used as the test bed in many studies, and thus tremendously promoted the research on automated dementia identification.

In this paper, we provide a survey of automated dementia identification approaches in the literature from a pattern classification perspective. Similar to other pattern classification solutions, various dementia identification approaches consist of two major steps: feature extraction and classification. Hence, we review the feature extraction methods and classifiers used in those approaches, respectively. We also provide a comparison of the reported performance of many available approaches.

## Methods

**Fig. 1:**
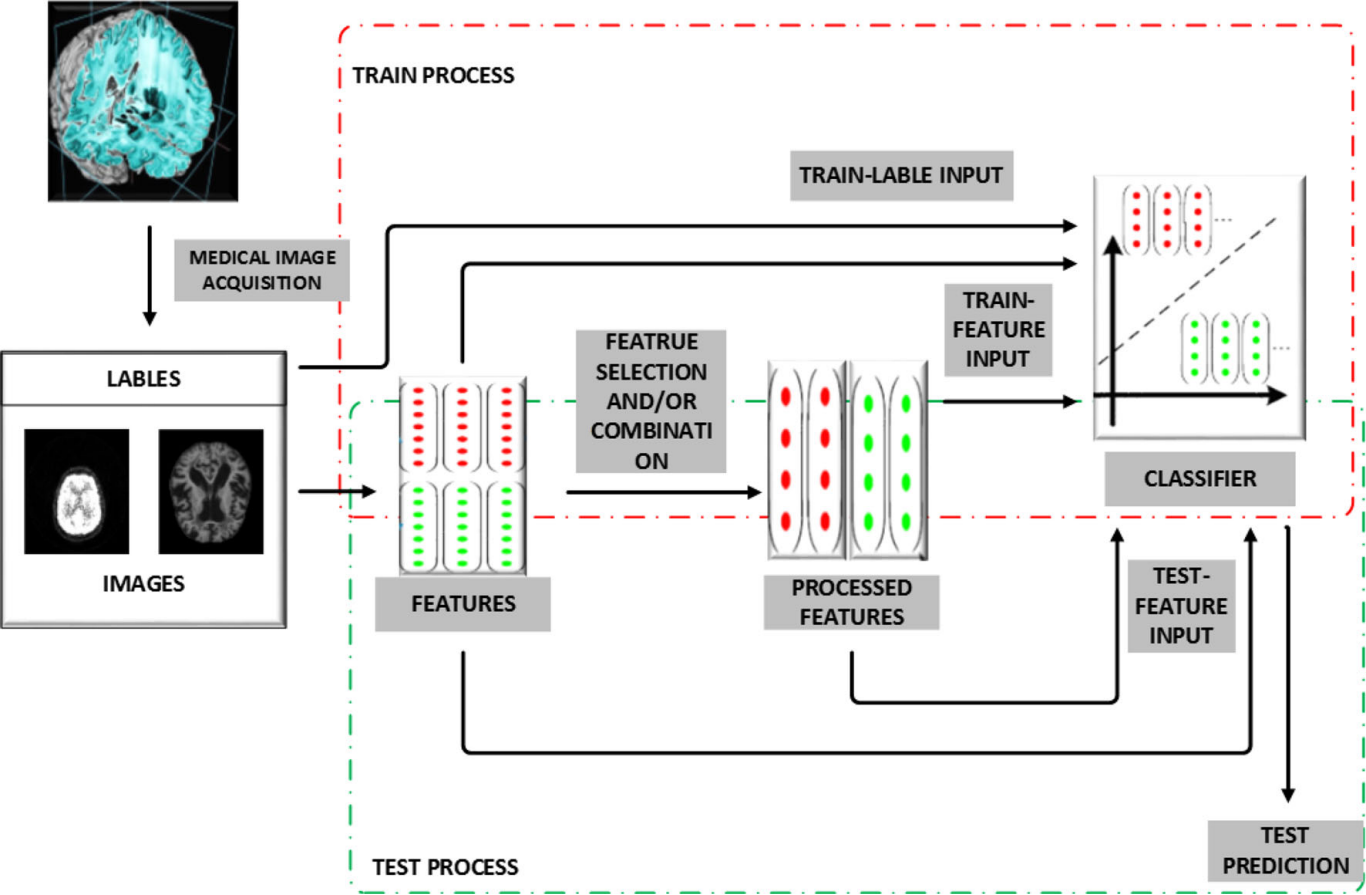
Diagram of computer-aided identification of dementia

Automated identification of dementia using medical imaging with the aid of computers is essentially an image-based pattern recognition problem, which can be solved in two successive steps: feature extraction and pattern classification. During the training stage, image features that can characterize the patterns of various types or stages of dementia are calculated based on the quantitative analysis of medical images. Those features are usually selected and/or combined to reduce their dimensionality before training a classifier with the supervised learning techniques [36]. The trained classifier may be treated as a “black box,” which encapsulates the knowledge gleaned from the images and is capable of producing the expected predictions [37]. For each testing image, the features extracted, selected, and combined in the same way are applied to the trained classifier to generate a predicted class label that indicates to which type or stage the dementia case belongs. The diagram of a typical automated dementia identification system is shown in Fig. 1.

Next, we will review the feature extraction methods and classification methods used in the state of the art dementia identification approaches, respectively.

### Feature extraction methods

According to the types of features extracted from brain images, feature extraction methods can be roughly grouped into voxel-based, vertex-based, and ROI-based ones [38].

#### Voxel-based methods

Voxel-based methods can be traced back to the mid-1990s, when Wright et al. [39] statistically analyzed the gray matter and white matter voxel values for schizophrenia diagnosis. Typically, voxel-based features consist of statistics of voxel distributions on major brain tissues, such as the gray matter, white matter, and cerebrospinal fluid (CSF) [37, 40–43]. Magnin et al. [44] counted the voxel value histogram in major anatomical regions, which could be obtained by either image segmentation or registering a brain atlas onto the image [44–46]. However, the anatomical parcellation of brain is not a trivial task and may not be adaptive to the pathology. Fan et al. [42] proposed an adaptive parcellation approach, in which the image space is divided into the most discriminative regions [40, 41, 47–49]. The voxel-based morphometry (VBM) method proposed by Ashburner et al. [50] allows investigation of focal differences in brain anatomy using the statistical parametric mapping (SPM), and hence greatly facilitates the extraction of voxel-based features. Papakostas et al. [51] successfully applied the VBM analysis to feature extraction on MRI data. Recently, Liu et al. proposed a simulation method to predict the longitudinal brain morphological changes in neurodegenerative brains based on VBM [52].

Voxel-based features can be either directly used to construct classifiers [43] or further processed to reduce its dimensionality via feature selection, agglomeration, and combination [38]. Vemuri et al. [37] used smoothing, voxel-downsampling, feature selection, and combination to identify the features with the highest discriminatory power. Zhao et al. [53] used the trace ratio linear discriminant analysis to get the optimal feature projection, and thus reduced the dimensionality of original features. Fan et al. [54] used a high-dimensional template to wrap original data and employed a watershed method to get the robust features.

#### Vertex-based methods

Clinical studies show that not only the volume of anatomical regions matters in the early diagnose of dementia, but also the vertex atrophy of the regions can reflect the difference among AD, NC, and MCI [55–57]. Hence, another category of features is defined at the vertex-level on the cortical surface. The cortical thickness represents a direct index of atrophy caused by dementia and can be used in dementia diagnosis. Querbes et al. [56] developed a fast, robust, and fully automated method for cortical thickness measurement. Lerch et al. [58] also proposed a link between histopathologically confirmed changes and cortical atrophy assessed through cortical thickness measurement. Desikan et al. [55] parcellated the brain into neocortical and non-neocortical ROIs by wrapping an anatomical atlas and used the mean thickness and volume of each ROI at the right and the left hemispheres as features. In this method, the volumes are corrected using the estimated total intracranial volume [38].

As an alternative to volumetric methods, cortical thickness measurement has given promising results while being less operator-dependent than the hippocampal volume measurements and is suitable for quantification and localization [59]. The cognitive reserve is recognized as a confounding factor in hiding early signs of dementia, especially for subjects with a high education level who would be more successful at coping with greater brain damage [60–62]. The studies, which have investigated the interaction between the cognitive reserve and neuroimaging modes, showed that neuroimaging measurements may reflect the underlying pathology better than neuropsychometry since they are less affected by cognitive reserve [61, 63–65]. However, clinical evaluations have shown the limitations of vertex-based features in predicting the evolution from the MCI stage to the dementia stage [66–69].

#### ROI-based methods

ROI-based methods define image features in one or more major brain components, such as the cingulum, corpus callosum, uncinate fasciculus, superior longitudinal fasciculus, and hippocampi. Pathological studies have shown that neurodegeneration in AD begins in the medial temporal lobe, successively affecting the entorhinal cortex, hippocampus, and limbic system, and then extends toward neocortical areas [70, 71]. There is a widespread agreement that medial temporal atrophy, in particular hippocampal atrophy, is a sensitive AD biomarker [72–74]. Hence, hippocampi have been used as a marker of early AD in a number of studies [38].

The widely used features include the volume or shape of hippocampi or a weighted combination. Chupin et al. [75–77] adopted the volume of the hippocampi as features, which were normalized by the total intracranial volume (TIV) summing up cortical parcellation maps of GM, WM, and CSF inside a bounding box in a standard space. Westman et al. [78] also used the hippocampal volume extracted on MRI data as features and the orthogonal partial least squares to latent structures (OPLS) analysis as the classifier to differentiate AD and MCI from elderly normal subjects. When it comes to the shape features, each segmented hippocampus is described by a series of spherical harmonics (SPHARM), whose coefficients were computed with the SPHARM-PDM software developed by the University of North Carolina and the National Alliance for Medical Imaging Computing [79]. Gerardin et al. [80] adopted two sets, one for each hippocampus, of 3D SPHARM coefficients as features and used an univariate feature selection method combined with a bagging strategy to get the most discriminative features. Atrophy in early stages of AD is not confined to the hippocampus or the entorhinal cortex. Other areas are affected in AD patients and MCI patients as well [4]. Therefore, multi-ROI-based feature extraction has attracted a lot of research attentions. Xia et al. [81] used the AAL cortical parcellation map to separate 116 anatomical regions for feature extraction. Liu et al. [82] proposed a multi-channel pattern analysis approach to analyze the hypo-metabolism patterns of AD and MCI on FDG-PET data and identified 21 brain regions as the most discriminative biomarkers.

In ROI-based methods, ROI segmentation is usually performed before feature extraction. Since manual segmentation is time-consuming and prone to operator-related bias, automated segmentation of ROIs is badly needed. Beside using probabilistic and anatomical priors for hippocampus segmentation [75], Chupin et al. [76, 77] also developed a fully automatic method called SACHA, which uses the prior knowledge on the location of the hippocampus and amygdala derived from a probabilistic atlas and on the relative positions of these structures with respect to the automatically identified landmarks [38]. The SACHA algorithm segments both the hippocampus and amygdala simultaneously based on competitive region-growing between these two structures. It has shown that this approach is competitive with manual tracing for the discrimination of patients with AD and MCI [75, 83].

### Classification methods

With the features estimated on training cases, a classifier can be trained and applied to predict the diagnosis of a testing case, whose features are extracted in the same way. The commonly used classifiers in dementia identification include the linear discriminant analysis (LDA), Bayes classifier, support vector machine (SVM), artificial neural network (ANN), and other supervised ones [84, 85].

#### Linear discriminant analysis (LDA)

Since the number of brain voxels is huge, the features calculated via voxel combination are of high dimension. LDA, also known as the Fisher linear discriminant (FLD), is one of the most popular dimensionality reduction methods [86]. LDA looks for low-dimensional linear combinations of variables, which best explain the data, by maximizing the between-class scatter matrix while minimizing the within-class scatter matrix and form a linear discriminant function resulting in least misjudgments [87–89]. Zhao et al. [53] proposed an improved iterative trace ratio (iITR) algorithm to solve the trace ratio linear discriminant analysis (TR-LDA) problem for dementia diagnosis and achieved better performance than the principal component analysis (PCA), locality preserving projections (LPP), and maximum margin criterion (MMC). Horn et al. [90] applied the image features compressed by the partial least squares (PLS) to LDA for differentiating AD from FTD and achieved an accuracy of 84 %, a sensitivity of 83 %, and a specificity of 86 % on perfusion SPECT images.

LDA is closely related to analysis of variance and regression analysis, which also attempt to express one dependent variable as a linear combination of other features or measurements [91]. LDA works well when the features own the characteristic of clear classification, which however is not possessed by most features extracted clinical data.

#### Bayes classifiers

Bayes classifiers are a family of simple probabilistic classifiers based on Bayes’ theorem with strong (naive) independence assumptions between the features. Seixas et al. [10] proposed a Bayesian network decision model for supporting diagnosis of AD, MCI, and NC, and achieved better performance than several well-known classifiers, including the näive Bayes, logistic regression model, multilayer perceptron ANN, decision table, decision stump optimized by the Adaboost algorithm and J48 decision tree. Liu et al. [92] proposed the multifold Bayesian Kernelization method, which can differentiate AD from NC with a high accuracy, but achieved poor results in diagnosing MCI-converter (MCIc) and MCI-non-converter (MCInc). Plant et al. [93] combined the feature selection with classification using a Bayes classifier for the discrimination between AD and NC on MRI data and reported an accuracy of up to 92 %. Lopez et al. [94] applied the multivariate methods, such as PCA and LDA, to feature extraction, and then employed the Bayesian framework for automated diagnosis of AD and NC using PET and SPECT.

#### Support vector machine (SVM)

A SVM constructs a hyperplane or a set of hyperplanes in a high- or infinite-dimensional space, which can be used for classification, regression, or other tasks [95]. Since the constructed hyperplane has the largest distance to the nearest training data points of any class, SVMs in general have lower generalization error than other classifiers, and hence have been commonly used to solve pattern classification problems which have limited training samples [38, 96–98]. Klöppel et al. [43] first used the SVM-based criteria to select the most discriminative features, and then applied the SVM-based classifier to diagnose healthy controls and schizophrenia patients using MRI brain images. Vemuri et al. [37] also used SVM as both feature selection criteria and a classifier, and achieved a sensitivity of 86 % and a specificity of 92 % in AD diagnosis on MRI data. Schmitter et al. [99] used SVM to verify that two distinct VBM algorithms, i.e., the FreeSurfer and an in-house algorithm MorphoBox, can achieve comparable results to the conventional whole-brain VBM techniques. Hackmack et al. [100] firstly used the dual-tree wavelet transform to extract features, and then used a linear SVM to discriminate multiple sclerosis from NC. Dukart et al. [101] used the meta-analysis- based-SVM to diagnose AD and NC on both MRI and PET data and achieved an accuracy of 90.0 %, a sensitivity of 91.8 % and a specificity of 87.8 %. Ortiz et al. [102] used the SVM classifier to verify the performance of three different feature extraction methods, including PCA, learning vector quantization (LVQ), and voxels as features (VAF) and demonstrated that LVQ features could generate the best result. Nir et al. [103] used the fiber-tract modeling method to extract image features and applied SVM to differentiating AD from NC and achieved an accuracy of 86.2 %, a sensitivity of 88.0 %, and a specificity of 89.2 %.

#### Artificial neural network (ANN)

ANNs are a family of models inspired by biological neural networks and are used to estimate or approximate functions that depend on a large number of inputs and are generally unknown. They have been used to solve a wide variety of tasks that are hard to solve using ordinary rule-based programming. Deng et al. [104] showed that using ANN can get higher sensitivity and accuracy than traditional discriminant function analysis [105] in dementia classification using MRI. Huang et al. [105] combined the VBM technique and ANN to differentiate AD from NC and achieved 100 % accuracy. García-Pérez et al. [106] employed the artificial neural network technology to build an automaton to assist neurologists during the differential diagnosis of AD and VD. The recent studies also suggest that deep learning, which is usually based on a hierarchical ANN, is effective in capturing high-level variations of brain images and improves the dementia classification [107–109].

Generally, ANN can be viewed as a ‘black box’ for the best discriminant analysis. Due to its parallel nature, ANN can easily take the advantage of hardware development and is typically suitable for solving classification problems with massive training data. However, tuning the parameters involved in ANN is often time-consuming, which has hampered the application of ANNs to dementia identification.

So far we have reviewed the application of four classical pattern classification methods to automated dementia identification. It is worth noting that dementia identification is essentially a supervised classification problem, and hence, the advances in supervised machine learning and pattern classification can find immediate application on this topic.

## Performance comparison

There are several comparative studies in the literature. Horn et al. [90] applied a set of 116 descriptors, which correspond to the average activity in ROIs calculated from the images of 82 AD and 91 FTD patients, to a number of linear and nonlinear classifiers, including the logistic regression, LDA, SVM, KNN, multilayer perceptron, and kernel logistic PLS. They compared the performance of those classifiers in differentiating AD from FTD and concluded that the PLS + KNN is the best method since it achieves the highest accuracy with leave-one-out cross-validation. Cuingnet et al. [38] evaluated the performance of ten approaches in automatically discriminating between patients with AD, MCI, and elderly controls using the T1-weighted MRI data acquired on 509 subjects from the ADNI database. In those approaches, the classifier is SVM and the involved feature extraction methods can be grouped into three categories. The first category is based on segmented tissue probability maps, including directly using the voxels of the tissue probability maps as features [43], using the STAND score [37], grouping the voxels into anatomical regions as features using a labeled atlas [44], and aggregating voxel values in homogeneously discriminative regions to form features [42]. The second category is based on the cortical thickness, including direct, atlas-based, and ROI-based methods. The third category is based on hippocampi, including the volume and shape of left and right hippocampus. They concluded that, for AD versus CN, whole-brain methods achieved high accuracies (up to 81 % sensitivity and 95 % specificity); for the detection of MCIc, the sensitivity was substantially lower; and for the prediction of conversion, no classifier obtained significantly better results than chance.

Next, we compare the performance of the automated dementia identification methods published in recent years in Table 1. It reveals that, when differentiating AD from NC, many methods can achieve an accuracy of >90 % and even 100 % on smaller datasets, whereas when separating MCI from AD or NC, the performance of those methods is much lower.

**Table 1:** Comparison of automated dementia identification methods in the literature

Year	Authors	Targets	Methods	Imaging modality	Data sets	Performance		
						Accuracy	Sensitivity	Specificity
2008	Klöppel et al. [43]	AD versus NC	VBM (GM) + SVM	MRI	34 AD versus 34 NC	95.6	97.0	94.1
2008	Xia et al. [81].	AD versus FTD versus NC	GA + MKL	FDG-PET	46 AD versus 43 FTD versus 40 NC	94.62	NaN	NaN
2007	Vemuri et al. [37]	AD versus NC	VBM +APOE + SVM	MR	190 AD versus 190 NC	89.3	86.0	92.0
2008	Magnin et al. [44]	AD versus NC	Histogram + SVM	MRI	16 AD versus 22 NC	94.5	91.5	96.6
2007	Fan et al. [42]	SC versus NC.	VBM + nonlinear SVM	MRI	Female: 23 schizophrenia versus 38 NC	90.2	NaN	NaN
					Male: 46 schizophrenia versus 41 NC	90.8	NaN	NaN
			VBM + linear SVM	MRI	Female: 23 schizophrenia versus 38 NC	88.5	NaN	NaN
					Male: 46 schizophrenia versus 41 NC	88.5	NaN	NaN
2009	Misra et al. [49]	MCI-C versus MCI-NC	VBM	MRI	ADNI	81.5	NaN	NaN
2009	Querbes et al. [56]	NC versus AD	Thickness-Atlas	MRI	ADNI: 30 AD versus 30 NC	85.0	NaN	NaN
2009	Desikan et al. [55]	MCI versus NC	Thickness-ROI	MRI	OASIS	91.0	73.0	94.0
					ADNI	91.0	94.0	85.0
2009	Gerardin et al. [80]	AD versus NC	Hippocampi shape + SVM	MRI	23 AD versus 23 MCI versus 25 NC	94.0	96.0	92.0
		MCI versus NC	Hippocampi shape + SVM	MRI	23 AD versus 23 MCI versus 25 NC	83.0	83.0	84.0
2009	Horn et al. [90]	AD versus FTD	PLS + LDA	SPECT	82 AD versus 91 FTD	84.0	83.0	86.0
			KL-PLS	SPECT	82 AD versus 91 FTD	84.0	80.0	87.0
			PLS + k-NN	SPECT	82 AD versus 91 FTD	88.0	93.0	85.0
			SVM	SPECT	82 AD versus 91 FTD	87.0	88.0	87.0
2013	Zhao et al. [53]	Dementia versus NC	KPCA + TR-LDA	–	289 demented versus 9611 NC	90.01	NaN	NaN
2008	Huang et al. [105]	AD versus NC	VBM + ANN	MRI	10 AD versus 12 NC	100	NaN	NaN
2010	Plant et al. [93]	AD versus NC	Data mining + SVM	MRI	32 AD versus 18 NC	90.0	96.88	77.78
		MCI versus NC	Data mining + Bayes	MRI	24 MCI versus 18 NC	85.71	83.33	88.89
2011	Westman et al. [78]	AD versus NC	OPLS	MRI	117 AD versus 122 MCI versus 112 NC	NaN	90.0	94.0
		AD versus MCI	OPLS	MRI	117 AD versus 122 MCI versus 112 NC	NaN	75.0	79.0
		MCI versus NC	OPLS	MRI	117 AD versus 122 MCI versus 112 NC	NaN	66.0	73.0
2012	Hackmack et al. [100]	MS versus NC	Wavelet transform + SVM	MRI	41 MS versus 26 NC	80.44	87.80	73.08
2013	Gray et al. [112]	AD versus NC	Random forest	MRI, PET	ADNI: 37 AD versus 35 NC	89.0	87.9	90.0
		MCI versus NC	Random forest	MRI, PET	ADNI: 75 MCI versus 35 NC	74.6	77.5	67.9
2013	Dukart et al. [101]	AD versus NC	Meta-analysis + SVM	MRI, PET	ADNI: 28 AD versus 28 NC	85.7	89.3	82.1
					Leipzig: 21 AD versus 13 NC	100.0	100.0	100.0
2013	Ortiz et al. [102]	AD versus NC	LVQ + SVM	MRI	ADNI: 25 AD versus 25 NC	91.0	90.0	88.0
			PCA + SVM	MRI	ADNI: 25 AD versus 25 NC	81.0	82.0	81.0
			VAF + SVM	MRI	ADNI: 25 AD versus 25 NC	71.0	76.0	66.0
2014	Nir et al. [103]	AD versus NC	Diffusion weighted method + SVM	MRI	ADNI: 37 AD versus 113 MCI versus 50 NC	86.2	88.0	89.2
		MCI versus NC				82.0	80.0	84.6
2015	Papakostas et al. [51]	AD versus NC	VBM + LC-kNN (k = 3)	MRI	OASIS: 49 mild AD versus 49 NC	80.0	80.0	79.0
			VBM + PNN	MRI	OASIS: 49 mild AD versus 49 NC	78.0	62.0	94.0
			DBM + LC-kNN (k = 3)	MRI	OASIS: 49 mild AD versus 49 NC	82.0	86.0	78.0
			DBM + Linear SVM	MRI	OASIS: 49 mild AD versus 49 NC	79.0	90.0	67.0
2015	Schmitter et al. [99]	AD versus NC	FreeSurfer + SVM	MRI	ADNI	NaN	82.0	88.0
			MorphoBox + SVM	MRI	ADNI	NaN	86.0	91.0
		MCI versus NC	FreeSurfer + SVM	MRI	ADNI	NaN	66.0	80.0
			MorphoBox + SVM	MRI	ADNI	NaN	69.0	83.0
2013	Liu et al. [92]	AD versus NC	Multifold Bayesian Kernelization	MRI, PET	ADNI: 85 AD versus 169 MCI versus 77 NC	84.74	NaN	NaN
		MCIc versus MCInc		MRI, PET		63.79	NaN	NaN
2009	Lopez et al. [94]	AD versus NC	PCA + Bayesian classifier	SPECT	38 AD versus 41 NC	88.6	NaN	NaN
				PET	42 AD versus 18 NC	98.3	NaN	NaN

## Perspective

Due to the advances in medical imaging, it is now possible to sequentially capture two separate yet complementary information of a patient study in a single scan, i.e., PET/CT [110]. Furthermore, it is predicted that the next-generation molecular imaging modalities will continuously advance in multi-modality paradigm, such as the recent development of PET/MRI and SPCET/CT [111]. Multimodal neuroimaging has several distinct advantages over single modality neuroimaging, including improving both spatial and temporal resolution, finding the anatomical basis for functional connectivity, targeting disease biomarkers with high specificity and sensitivity, along with many new opportunities to improve brain research [109]. Recently, Gray et al. [112] proposed a multi-modality classification framework, in which manifolds are constructed based on pairwise similarity measures derived from random forest classifiers, and achieved classification accuracies of 89 % between AD and NC, and 75 % between MCI and NC. Liu et al. [113] summarized the recent advances in multimodal neuroimaging technologies, along with their applications to the neuropsychiatric disorders. We believe that the application of multi-modality neuroimaging will substantially improve the performance of automated dementia identification.

## Conclusion

In this paper, we provide a brief review of automated dementia identification algorithms, which from a pattern classification perspective can be divided into two stages: feature extraction and classification. We summarize the voxel-based, vertex-based, and ROI-based feature extraction methods and LDA-based, Bayesian, SVM-based, and ANN-based pattern classification methods used in various dementia identification algorithms. We also compare the performance of some of those algorithms. The comparison shows that satisfying diagnosis of AD and NC can be achieved by many algorithms; whereas differentiating MCI from AD or NC still remains a major challenge. Therefore, more research effort should be devoted to discovering the patterns embedded in brain images of MCI patients. We expect novel solutions could be proposed to address this issue.
